# Review of Biomass-Derived Carbon Nanomaterials—From 0D to 3D—For Supercapacitor Applications

**DOI:** 10.3390/nano15040315

**Published:** 2025-02-19

**Authors:** Yihong Yan, Weiqiang Sun, Yuxin Wei, Kuankuan Liu, Jingjing Ma, Guang Hu

**Affiliations:** 1 School of Nuclear Science and Technology, Xi’an Jiaotong University, Xi’an 710049, China; yihong@stu.xjtu.edu.cn (Y.Y.); sunweiqiang@xjtu.edu.cn (W.S.); 2 School of Human Settlements and Civil Engineering, Xi’an Jiaotong University, Xi’an 710049, China; weiyuxin5924@stu.xjtu.edu.cn (Y.W.); 15940561336@163.com (K.L.); jingjma2022@163.com (J.M.)

**Keywords:** biomass, nanomaterials, supercapacitors, porous carbon

## Abstract

The transition to sustainable energy storage solutions has driven significant interest in supercapacitors, which offer high power density, rapid charge–discharge capabilities, and exceptional cycle stability. Biomass-derived carbon nanomaterials have emerged as compelling candidates for supercapacitor electrodes due to their renewable origins, environmental compatibility, and cost-effectiveness. This study explores recent advancements in tailoring structural properties, for example in preparation methods and activation, which are essential for efficient charge storage and rapid ion transport. Attention is given to the dimensional configurations—spanning 0D to 3D structures—and their impact on electrochemical behaviors. This review outlines the challenges faced in scaling up and optimizing these materials for practical applications, alongside an outlook on future research directions. By bridging the gap between material design and application demands, this work contributes to advancing sustainable supercapacitor technologies for a greener energy future.

## 1. Introduction

In the global context of rapidly advancing technology, energy storage technologies play an increasingly crucial role in addressing growing energy demands and environmental challenges. Traditional energy storage devices, such as lithium-ion batteries, exhibit high energy density; however, they suffer from limited power density, slow charge–discharge rates, and relatively short cycle life [1]. These limitations restrict their application in scenarios that require fast charging–discharging capabilities and long operational lifespans. Consequently, supercapacitors (also known as electrochemical capacitors) have garnered significant attention as emerging energy storage devices [2].

Supercapacitors, known for their high power density and long cycle life, operate through electric double-layer capacitance (EDLC) and Faradaic types (pseudocapacitance and battery-type behavior) [3]. EDLCs store energy via ion adsorption at the electrode–electrolyte interface, while pseudocapacitors rely on reversible Faradaic reactions for charge storage, enabling rapid energy transfer. The performance of supercapacitors is highly dependent on electrode materials, with carbon-based materials (e.g., activated carbon, graphene, carbon nanotubes) being widely used in EDLCs due to their high surface area, good conductivity, and low cost [4]. Metal oxides (e.g., RuO_2_, MnO_2_) and conductive polymers (e.g., polyaniline, polypyrrole) [5] offer higher specific capacitance through pseudocapacitance but face challenges such as high production costs, energy-intensive fabrication, and environmental impact. Materials like graphene and RuO_2_, while high-performing, require complex and costly synthesis processes, limiting their commercial viability and raising sustainability concerns. To address these challenges, hybrid EDLC and Faradaic mechanisms have gained increasing attention [6]. Conductive carbon materials, particularly biomass-derived carbon, are ideal substrates for such electrodes. These materials offer natural porosity, high surface area, and renewable origins, making them excellent candidates for energy storage. Carbon’s conductive properties facilitate efficient charge transfer, while its porous structure provides abundant nucleation sites for pseudocapacitive materials, enhancing overall performance. These materials offer cost-effective, sustainable alternatives to traditional carbon-based electrodes, with low energy consumption during production and efficient utilization of waste resources. Biomass-derived carbons demonstrate excellent capacitance, energy density, and cycling stability, making them highly suitable for sustainable supercapacitor applications [7,8,9].

The development of biomass-derived carbon materials for supercapacitor applications has progressed significantly, with a focus on optimizing porosity, enhancing conductivity, and improving overall electrochemical performance [10,11,12]. Advanced activation methods have enabled precise control over hierarchical porous structures, facilitating ion diffusion and charge storage, while heteroatom doping with elements like nitrogen, sulfur, and phosphorus has further boosted conductivity and pseudocapacitance [13]. Researchers have also combined biomass-derived carbons with transition metal oxides and conductive polymers to create hybrid materials that leverage synergistic effects for higher capacitance and energy density [14]. Dimensional engineering, ranging from 0D spheres to 3D porous networks, has been instrumental in optimizing charge transfer and ion transport. For instance, 0D spheres provide abundant charge storage sites, 1D fibers enable rapid ion and electron transport, 2D nanosheets offer extensive surface areas with active edges, and 3D porous structures integrate continuous pathways for efficient ion diffusion and electron conduction.

In this review, we focus on summarizing the effects of carbon material dimensions on the performance of supercapacitors, while also discussing the influence of preparation methods on their structural and chemical properties. The dimensionality of carbon materials—such as 0D spheres, 1D fibers, 2D nanosheets, and 3D porous networks—is primarily determined by precursor selection and structural design strategies, including template methods and self-assembly. However, pyrolysis processes and activation strategies also play a crucial role in shaping the microstructure, porosity, surface area, and conductivity of the materials, which indirectly impact their dimensional features and overall performance. This review systematically examines how these dimensional configurations influence the electrochemical behavior of supercapacitors. By analyzing the interplay between preparation methods, material dimensions, and their effects on supercapacitor performance, this work aims to provide comprehensive insights into optimizing carbon materials for scalable and high-performance energy storage applications.

## 2. Charge Storage Mechanisms of Supercapacitors

Supercapacitors are an emerging type of energy storage device that bridge the gap between traditional capacitors and chemical batteries. Their unique charge storage mechanisms endow them with the advantages of high power density and long cycle life. The charge storage in supercapacitors mainly relies on three mechanisms: EDLC, pseudocapacitance, and battery-type behavior [15].

The EDLC is based on the principle of physical adsorption [16]. When an external electric field is applied to the interface between the electrode and the electrolyte, ions in the electrolyte form a double-layer structure at the electrode surface. One layer consists of ions adsorbed on the electrode surface, while the other layer is the diffuse layer of counter-ions in the electrolyte. This process does not involve chemical reactions, and the charges are stored electrostatically within the double layer. Since the thickness of the electric double layer is typically at the nanometer scale, and its capacitance is closely related to the specific surface area of the electrode material, high-specific-surface-area porous materials, such as activated carbon, carbon nanotubes, and graphene, are commonly used to enhance energy storage performance [17]. The advantage of EDLC lies in its high power density and long cycle life, but its energy density is relatively low.

In contrast, pseudocapacitance relies on Faradaic reactions occurring on the surface of the electrode material [18]. These reactions enable charge storage through the transfer of electrons and ions during electrochemical processes. Common pseudocapacitive materials include transition metal oxides and conductive polymers [19], which offer high charge storage capacities through multi-electron transfer. Transition metal oxides typically show specific capacitance in the range of 200–1200 F/g, while conductive polymers generally have specific capacitance values ranging from 200–800 F/g. These materials can achieve higher charge storage capacities through multi-electron transfer during electrochemical reactions. Compared with EDLC, pseudocapacitance provides significantly higher energy density but may have slightly lower power density and cycle life. Additionally, battery-type behavior refers to the charge storage mechanism in which ions intercalate or deintercalate within the electrode material, akin to the processes seen in batteries. This mechanism is diffusion-controlled and typically involves materials such as metal oxides or sulfides. Battery-type electrodes contribute to even higher energy densities compared to EDLC and pseudocapacitors, but they often come with the drawback of lower power density and limited cycle stability due to the diffusion process.

## 3. Preparation Methods for Biomass-Derived Porous Carbon Materials

The preparation of biomass-derived porous carbon materials involves converting natural biomass into carbon-rich materials with well-developed porosity and tailored surface properties. The process typically consists of two key stages: carbonization and activation, which can be performed using various techniques to achieve materials with specific characteristics suitable for supercapacitor applications.

### 3.1. Carbonization Process

**Figure 1:**
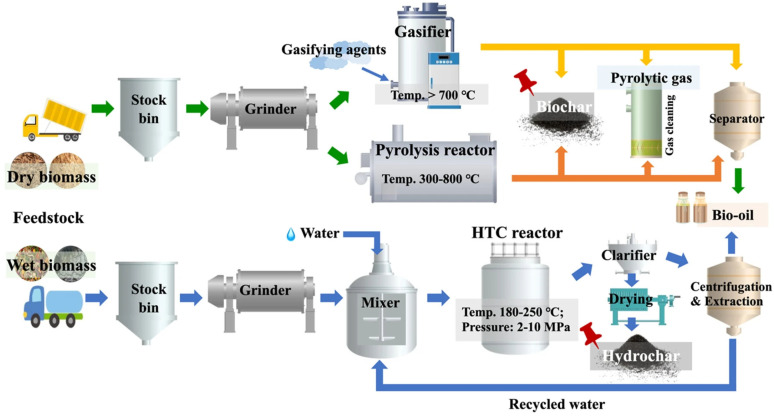
Three biomass conversion processes for biochar production: pyrolysis, gasification, and hydrothermal carbonization [21]. Note: HTC: hydrothermal carbonization.

Carbonization is the process of heating organic materials to induce thermal decomposition, leaving behind carbon or carbonaceous residues. It is an essential step in preparing porous carbon materials from biomass [20]. During carbonization, most non-carbon elements in the raw material are removed, producing carbonized materials with initial porosity, mechanical strength, and a fixed-carbon framework, which lays the foundation for subsequent activation processes. Current research on carbonization focuses on producing graphitized carbon with developed initial porosity, primarily pyrolysis, gasification, and hydrothermal carbonization (HTC) (Figure 1). Each method differs significantly in terms of feedstocks, operational conditions, and the physicochemical properties of the resulting biochar. Below, the preparation processes and key comparisons of these methods are outlined in Table 1.

#### 3.1.1. Pyrolysis Biochar

Pyrolysis is the most commonly employed method for biochar production due to its simplicity and economic feasibility for large-scale applications. This process involves heating biomass in an oxygen-limited environment at temperatures ranging from 300 to 800 °C [22]. The slow heating rate (typically 5–10 °C/min) and residence time (often exceeding one hour) allow for controlled thermal decomposition, leading to the formation of carbon-rich biochar, along with co-products such as bio-oil and syngas. In battery applications, the biochar produced through pyrolysis has been shown to possess high surface area and electrical conductivity, making it suitable for use an electrode material in supercapacitors and lithium-ion batteries. The controlled pore structure formed during pyrolysis enhances the charge storage capacity, which is crucial for improving the energy and power density of energy storage devices [23].

Pyrolysis temperature is a critical factor that influences the physicochemical properties of the biochar. At lower temperatures, biochar has a higher yield but lower specific surface area and porosity. As the temperature increases, carbon stability improves, and pore volume expands due to the removal of pore-blocking substances and the transformation of carbon phases from amorphous to graphitic. For instance, biochar derived from sawdust exhibits a 3.9-fold increase in surface area when pyrolysis temperature is raised from 400 to 700 °C [24]. Similarly, wood waste-derived biochar can achieve a 29.5-fold increase in surface area when pyrolyzed at 950 °C compared to 650 °C [25]. The longer residence time promotes the development of a porous structure and enhances biochar quality. These properties are particularly beneficial for enhancing the conductivity and cycling stability of electrodes in supercapacitors and batteries [26,27].

#### 3.1.2. Gasification Biochar

Gasification is a thermochemical process that operates at higher temperatures (above 700 °C) than pyrolysis and involves the presence of gasifying agents such as air, O_2_, CO_2_, or steam. The process consists of four distinct stages: drying (100–200 °C), pyrolysis (200–700 °C), combustion (700–1500 °C), and reduction (800–1000 °C) [28]. While the primary aim of gasification is to produce syngas (a mixture of CO, H_2_, CO_2_, and CH_4_), biochar is obtained as a by-product. Gasification biochar is of interest for energy storage applications due to its high surface area and microporosity, which are ideal for use in electrodes for supercapacitors and lithium-ion batteries. The increased surface area from gasification enhances ion adsorption and reduces the internal resistance of supercapacitors, improving their energy and power density [29].

The high-temperature environment and use of gasifying agents promote the release of volatiles, resulting in biochar with high aromaticity and well-developed microporous and mesoporous structures. In the context of batteries, gasification biochar’s structural properties enable fast ion diffusion, making it an excellent candidate for an anode material in high-performance batteries. The feedstock’s ash content and the presence of alkali and alkaline earth metals (AAEMs) significantly affect gasification. High-ash feedstocks like sewage sludge and digestates undergo solid–solid interactions between carbon and AAEMs, weakening carbon–carbon bonds and enhancing biochar reactivity [30]. This reactivity can be advantageous in lithium-ion batteries, where the interaction of biochar with electrolytes can enhance the overall electrochemical performance.

Compared to pyrolysis biochar, gasification biochar typically has a lower yield due to the higher release of volatiles but exhibits superior pore structure and specific surface area, making it an excellent choice for applications requiring high porosity. Gasification biochar is characterized by a highly porous structure, with increased micro- and mesoporosity and a larger surface area, which enhances its adsorption and catalytic properties. This makes gasification biochar a promising material for enhancing the cycling stability and performance of energy storage devices such as supercapacitors and lithium-ion batteries.

#### 3.1.3. Hydrothermal Carbonization Biochar

HTC is a distinctive method that operates under subcritical water conditions at moderate temperatures (180–250 °C) and pressures (2–10 MPa) [31]. Unlike pyrolysis and gasification, HTC can process wet and bulky biomass without requiring energy-intensive drying, making it particularly suitable for feedstocks such as food waste, yard waste, and wastewater sludge [32]. During HTC, water acts as a reaction medium, significantly enhancing hydrolysis, dehydration, and polymerization reactions. Hydrochar produced from HTC has gained attention in battery applications due to its high oxygen content, which can improve the electrochemical reactivity and surface functionality of biochar electrodes in supercapacitors and batteries.

The HTC process involves the decomposition of carbohydrates, proteins, lipids, and lignin in biomass into intermediates through hydrolysis, decarboxylation, and deamination. These intermediates then undergo aromatization and polymerization to form hydrochar. While HTC produces the highest biochar yield among the three methods, the resulting hydrochar typically has lower graphitization and porosity compared to pyrolysis and gasification biochars [33]. However, hydrochar retains more oxygenated functional groups, which can be advantageous for applications requiring high surface reactivity. In the context of batteries, the oxygenated groups present in hydrochar can enhance the interface interaction between the electrode material and the electrolyte, improving the cycling stability and charge–discharge efficiency of batteries [34]. HTC also allows for the preservation of nutrients and minerals, but it may result in the accumulation of undesirable substances such as polycyclic aromatic hydrocarbons, which must be carefully addressed before hydrochar can be used in practical applications. Further activation or carbonization of HTC-derived hydrochar can improve its porosity and surface area, thus enhancing its performance as an anode material in lithium-ion and sodium-ion batteries [35].

### 3.2. Activation Methods

The activation of biomass-derived carbon materials plays a crucial role in enhancing their electrochemical performance, especially for applications in energy storage devices [36]. The primary aim of activation is to increase the specific surface area (~1000 m^2^/g), porosity (high micro- and mesoporosity), and surface functionality of the carbon material, all of which are essential for improving charge storage capacity, ion diffusion rate, and overall electrochemical stability. The two main activation methods are physical activation and chemical activation, both of which are used to tailor the structure and properties of carbon materials for battery applications [37].

#### 3.2.1. Physical Activation

**Figure 2:**
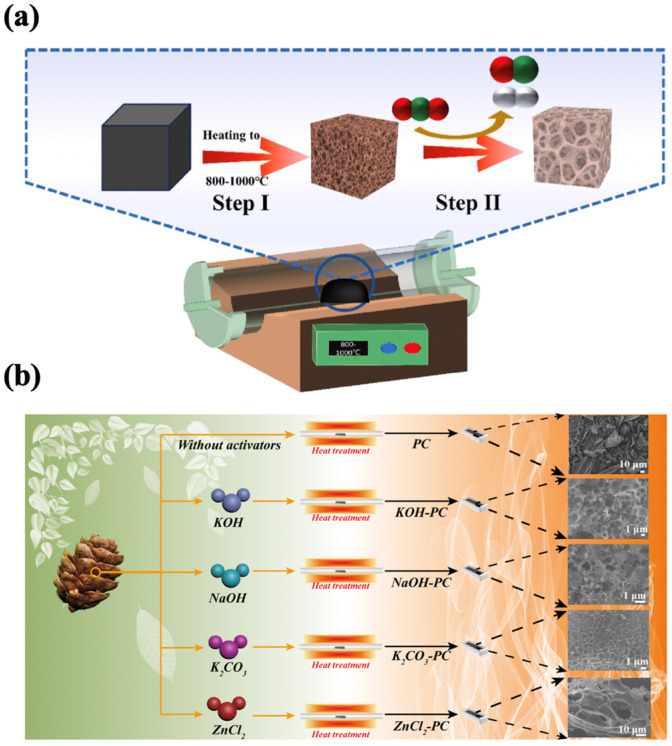
(**a**) Simple schematic drawing of physical activation [43]. (**b**) Schematic illustration of the preparation of porous carbon materials with different activators [44].

Physical activation is a widely used method for enhancing the porous structure of biomass-derived carbon materials, which is essential for improving their performance in supercapacitors. The process involves two key steps: first, pyrolyzing the biomass precursor at temperatures between 450 and 900 °C in an inert atmosphere (e.g., nitrogen or argon) to form a carbon material; and second, exposing the resulting carbon to oxidizing gasses such as CO_2_, steam, or their mixtures at higher temperatures (600–1200 °C) (Figure 2a). This process is highly effective in creating a well-developed pore structure, which significantly improves the material’s surface area, porosity, and electrochemical properties. During physical activation, the carbonized material reacts with oxidizing gasses, leading to partial etching of the carbon surface and the creation of pores. For instance, in steam activation, water vapor reacts with the carbon surface to form carbon oxides such as CO_2_ or CO, which can further react with the carbon material, resulting in the generation of micropores and mesopores. The process typically requires high temperatures (above 750 °C) to drive these reactions, but care must be taken not to exceed 900 °C, as excessively high temperatures can lead to inefficient pore formation. A balanced temperature allows the steam to diffuse into the carbon material and efficiently etch the surface, resulting in a more uniform pore structure.

Steam activation has gained attention due to its simplicity, low operation difficulty, and environmental friendliness. For example, Ima et al. [38] used a two-stage steam activation process to produce mesoporous activated carbon (AC) from needle coke, achieving a maximum specific surface area (SSA) of 1134 m^2^/g. Similarly, Selvaraju et al. produced micro-mesoporous AC from Artocarpus integer bio-waste, with an SSA of 1150.12 m^2^/g, through steam activation at around 850 °C [39]. The steam activation process not only increases surface area but also affects the pore volume and morphology of the resulting material, which are critical for optimizing electrochemical performance in supercapacitors and lithium-ion batteries. Son et al. [40] employed an adjusted steam activation strategy, controlling steam pressure, activation time, and temperature, and achieved an SSA of 2351 m^2^/g with a total pore volume of 1.61 cm^3^/g, indicating the high potential of steam activation for producing high-quality carbon materials.

In addition to steam activation, CO_2_ activation is another widely used method, particularly for producing carbon materials with a high specific surface area. CO_2_ reacts with the carbon material at elevated temperatures (850 to 1100 °C) to create micropores, a process that is slower than steam activation but easier to control. The reaction between CO_2_ and carbon can be summarized as

(1) $${CO}_{2}+C\to 2CO$$

(2) $$2CO+O_{2}\to 2{CO}_{2}$$

This reaction leads to the gradual etching of the carbon surface, forming additional micropores that enhance the material’s surface area. CO_2_ activation is considered environmentally friendly, as CO_2_ is a naturally occurring, low-cost, and non-toxic gas. Gunasekaran et al. [41] demonstrated that CO_2_ activation could yield meso- and macroporous carbon materials from hemp fiber, achieving an SSA of 1060 m^2^/g. Similarly, Jiang et al. [42] used CO_2_ activation to convert lignocellulosic biomass into hierarchical porous carbon with an SSA of up to 738.74 m^2^/g at 800 °C. CO_2_ activation’s slower rate allows for better control over pore structure, making it an attractive method for producing carbon materials with well-defined mesoporous structures, which are crucial for battery applications.

The advantages of physical activation include its simplicity, low cost, and lack of pollution, making it an attractive method for large-scale production. However, one of the challenges of physical activation is that it often requires higher temperatures and longer activation periods to achieve a large specific surface area and extensive pore networks. Furthermore, while physical activation is effective at creating pores, it may not always achieve the high surface area or extensive mesopore networks required for some advanced energy storage applications. In summary, physical activation is a cost-effective and environmentally friendly method that can significantly enhance the electrochemical properties of biomass-derived carbon materials, particularly for use in supercapacitors and lithium-ion batteries. The process can be further optimized by adjusting activation parameters such as temperature, activation time, and the choice of oxidizing agent, which influences the surface area, pore structure, and overall performance of the carbon materials. While physical activation remains an essential method for improving carbon materials, ongoing research and development are focused on overcoming its limitations, such as optimizing reaction times, reducing energy consumption, and improving pore structure uniformity.

#### 3.2.2. Chemical Activation

Chemical activation is a highly effective method for enhancing the surface area, porosity, and functionalization of biomass-derived carbon materials, making them particularly suitable for energy storage applications such as supercapacitors and lithium-ion batteries. Chemical activation, using activators like H_3_PO_4_, KOH, and ZnCl_2_ at 300–950 °C, offers controllable reactions and diverse porous carbon morphologies from biomass (Figure 2b). The impregnated material is then carbonized at high temperatures (typically 450–1000 °C) in an inert gas environment [37]. During the activation process, the chemical agents act as dehydrating agents, promoting the removal of volatile substances, while simultaneously oxidizing and etching the carbon framework to create a well-developed porous structure.

Among the various chemical activators, KOH is the most widely used and effective reagent due to its ability to create extensive micropores and mesopores with high specific surface areas. The activation mechanism begins with the reaction between KOH and carbon at temperatures around 400–600 °C, forming potassium carbonate (K_2_CO_3_) and potassium oxide (K_2_O) [45]. At higher temperatures (>762 °C), metallic potassium vapors intercalate into the carbon lattice, leading to expansion, twisting, and the formation of new pore channels. This results in the generation of a hierarchical porous structure that is critical for improving ion transport and charge storage in energy storage devices [46]. For example, KOH-activated carbon derived from tea waste demonstrated a high surface area of 1610 m^2^/g with a hierarchically microporous structure, leading to enhanced electrochemical performance [47]. Similarly, biomass waste such as bamboo chips and squid gladius chitin has been successfully activated with KOH to yield high-surface-area carbons with oxygen and nitrogen doping, improving both conductivity and energy density in supercapacitors [48]. In another study, grape marc carbon impregnated with KOH and nitrogen-doped with urea showed a surface area of 1356 m^2^/g, with a hierarchical pore structure, achieving a specific capacitance of 139 F/g in NaOH electrolyte. This highlights the effectiveness of nitrogen doping in improving charge transfer and chemisorption of oxygen species, demonstrating the potential of KOH-activated, nitrogen-doped carbons for high-performance energy storage [49]. Activated carbon from Australian hemp hurd, synthesized with KOH activation, showed a capacitance of 240 F/g at 1 A/g in Na_2_SO_4_ electrolyte. When combined with electrolytic manganese dioxide, the hybrid capacitor achieved 95 F/g capacitance and 38 Wh/kg energy density, illustrating the benefits of KOH activation for enhanced electrochemical performance [50]. Moreover, KOH treatment significantly enhanced the surface area and pore structure of walnut septum-derived porous carbon, improving its specific capacitance and performance in supercapacitors [51].

In addition to KOH, NaOH serves as a cost-effective alternative for chemical activation. NaOH activation tends to produce a higher proportion of mesopores, which are advantageous for facilitating ion diffusion and electrolyte penetration in electrochemical systems [52]. NaOH-activated carbons have been reported to achieve specific surface areas exceeding 3000 m^2^/g with hierarchical pore structures, demonstrating excellent capacitance and cycling stability in supercapacitors [53]. Furthermore, NaOH activation minimizes the generation of hazardous by-products compared to KOH, offering greater industrial scalability and environmental compatibility.

H_3_PO_4_ is another commonly employed chemical activator, particularly for lignocellulosic biomass precursors [54]. The activation process begins with the impregnation of H_3_PO_4_ into biomass, where it hydrolyzes and interacts with the lignin and cellulose components, resulting in the formation of polyphosphates. These polyphosphates further react at high temperatures, promoting the development of a uniform microporous and mesoporous structure. H_3_PO_4_ activation also introduces oxygen-rich functional groups (e.g., –OH, –COOH) that enhance the surface reactivity of the carbon material [55]. For example, walnut shell-derived carbon activated with H_3_PO_4_ achieved a surface area of 2583 m^2^/g with rich heteroatom doping, making it highly suitable for high-performance energy storage applications [56]. In addition, the introduction of phosphorus and oxygen heteroatoms into the natural honeydew peel chemical structure has shown to be an effective method for synthesizing activated carbon with high-performance energy storage potential [57]. H_3_PO_4_ activation of honeydew peel results in the formation of phosphorylation with cellulose fibers, which helps stabilize the cellulose structure and prevents significant degradation during processing. This heteroatom doping significantly enhances the electrochemical properties of the activated carbon, leading to improved specific capacitance and retention, making it highly suitable for supercapacitor applications.

Another one is ZnCl_2,_; while it has a long history in chemical activation, its usage has declined due to environmental concerns regarding hazardous wastewater generated during the washing process [58]. Nevertheless, ZnCl_2_ is effective in facilitating the formation of well-developed micropores through its role as a dehydrating agent and catalyst. Carbons activated with ZnCl_2_ typically exhibit high surface areas and uniform pore distributions, which are beneficial for adsorption and energy storage applications [59]. A significant advantage of chemical activation over physical activation is its ability to produce carbons with higher specific surface areas, well-developed pore structures, and adjustable pore size distributions [60]. The chemical activation process also allows for the incorporation of heteroatoms (e.g., nitrogen, sulfur, phosphorus) into the carbon framework, further enhancing the material’s electrochemical properties. For instance, ternary-doped carbon nanosheets with oxygen, nitrogen, and sulfur functional groups have been synthesized via KOH activation at 600 °C, achieving a specific surface area of over 1000 m^2^/g and excellent electrochemical performance [61]. Despite its advantages, chemical activation faces certain challenges, such as the requirement for extensive washing to remove residual activators and the generation of hazardous wastewater. These issues must be addressed through optimized processes to improve environmental sustainability and reduce production costs. In recent years, microwave-assisted chemical activation has emerged as an innovative approach, enabling rapid and uniform heating while reducing energy consumption and activation time [62]. Combining physical and chemical activation strategies has also been explored to create carbon materials with synergistically enhanced properties, such as hierarchical pore structures and improved surface chemistry.

In summary, both physical and chemical activation methods play essential roles in optimizing the properties of biomass-derived carbon materials for energy storage devices. Physical activation is highly effective in increasing the surface area and porosity of the carbon material, which is beneficial for enhancing the power density and energy storage capacity in supercapacitors and improving the performance of lithium-ion batteries. On the other hand, chemical activation introduces functional groups that significantly improve the reactivity and electrochemical performance of the material, making it suitable for applications where high capacity and long cycle life are critical. The choice of activation method depends on the specific requirements of the battery application, with physical activation being favored for applications requiring high power density and chemical activation being preferred for those needing high energy density and stability.

#### 3.2.3. Effect of Pore Structure and Doping

To further understand the electrochemical performance of biomass-derived carbon materials, it is essential to consider the impact of pore structure and surface functional groups. These factors, closely related to the activation process, significantly influence the overall performance of the materials in energy storage applications. During activation, the formation of macro-, meso-, and micropores plays a critical role in optimizing ion transport and charge storage. Macropores and mesopores create efficient channels for ion diffusion, enhancing the power density and enabling rapid charge/discharge rates. Meanwhile, micropores contribute primarily to the specific capacitance by increasing the surface area available for charge storage, particularly at lower current densities.

In addition to pore structure, the surface functional groups formed during the activation process also contribute to electrochemical performance. Groups such as hydroxyl, carboxyl, and carbonyl enhance the material’s wettability and facilitate better electrolyte interaction. The doping of heteroatoms, particularly nitrogen, further boosts electrochemical behavior. Nitrogen doping increases pseudocapacitance by providing additional charge storage sites and improving the material’s surface reactivity, which leads to better conductivity and overall energy storage performance [63]. When combined with other heteroatoms, such as boron or sulfur, nitrogen doping can induce synergistic effects, further optimizing the material’s electrochemical properties and making it more suitable for high-performance supercapacitors. The influence of nitrogen doping and other heteroatom incorporations will be discussed in detail in Section 4.

## 4. Versatile Dimension of Biomass-Based Carbon and Its Application in Supercapacitors

Biomass-derived carbon materials possess diverse microstructures, from 0D to 3D, which inherit and enhance the natural advantages of the original biomass [64]. Different dimensional structures—zero-dimensional (0D) carbon spheres, one-dimensional carbon (1D) fibers, two-dimensional (2D) nanosheets, and three-dimensional (3D) porous networks—offer unique advantages in optimizing ion diffusion, electron transport, and charge storage mechanisms. Zero-dimensional carbon spheres offer high surface area and uniform charge distribution, ideal for energy storage. One-dimensional fibers and tubes promote efficient ion transport, reducing resistance. Two-dimensional carbon sheets contribute to high conductivity and increased surface area for better charge storage. Three-dimensional honeycomb-like structures optimize ion accessibility and shorten ion diffusion paths, making them suitable for high-performance supercapacitors. Each dimension offers specific benefits that enhance the material’s electrochemical properties. The following section explains each dimension in detail, and Table 2 summarizes some of the electrochemical properties.

### 4.1. OD Biomass-Based Carbon

**Figure 3:**
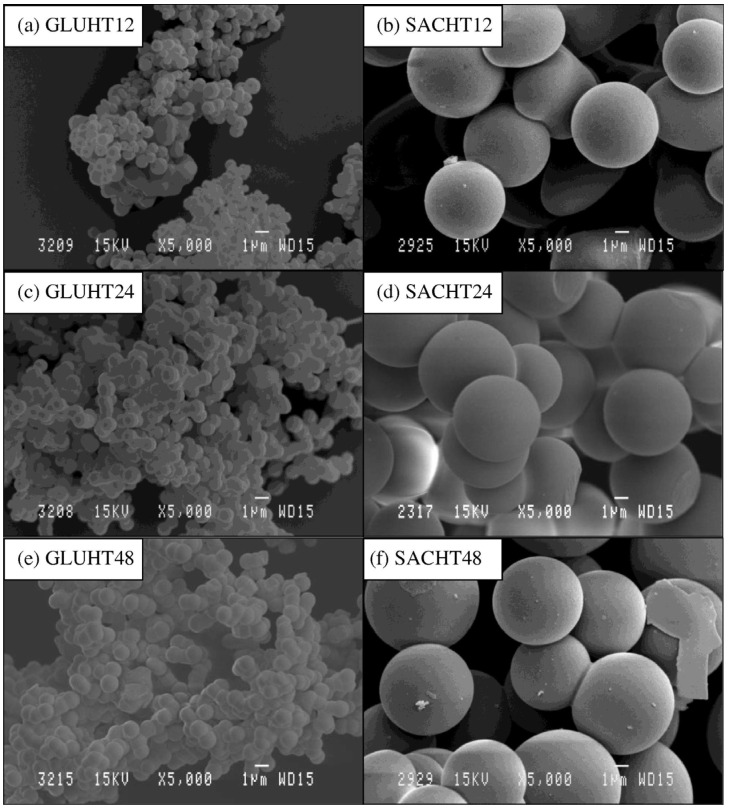
SEM images of the spherical carbons obtained by hydrothermal treatment of glucose and saccharose at different times: (**a**,**b**) at 12 h, (**c**,**d**) at 24 h, (**e**,**f**) at 48 h [85].

Zero-dimensional materials are structures where all three dimensions are confined to the nanoscale, typically below 100 nm. These materials lack any extended length, width, or height, making them effectively “point-like” at the nanoscale. In the context of biomass-derived carbon materials, 0D materials are highly attractive due to their high surface-to-volume ratio, which provides abundant active sites for charge storage and surface reactions. Biomass-derived 0D carbon nanostructures, such as carbon nanospheres, have attracted significant interest due to their excellent chemical stability, rich surface chemistry, and easy modification capabilities [83]. These materials are typically synthesized from sugar-based precursors, including sucrose, glucose, xylose, and starch, using the hydrothermal method [84]. Figure 3 shows the 0D spherical carbon from glucose and saccharose. The resulting 0D spherical carbon materials often possess smooth surfaces, and the aggregation or stacking of these carbon spheres leads to the formation of abundant void structures. These voids not only provide substantial charge storage space but also significantly enhance the ion-accessible surface area, making these materials highly advantageous for applications in adsorption, energy storage, and catalysis.

For example, lignin extracted from corn cobs has been used as a biomass precursor to prepare hollow spherical carbon materials through spray drying and carbonization techniques [65]. These hollow carbon nanospheres exhibit a well-developed micro/mesoporous structure that is particularly suited for use as supercapacitor electrode materials. The hollow cavities in the nanospheres act as efficient reservoirs for electrolyte storage, while the interconnected micro/mesopores provide ample ion storage sites and rapid ion transport pathways. This unique structure enables the material to deliver high power density and excellent rate performance, which are critical for high-performance supercapacitors. In addition, Jiang et al. [66] developed carbon microsphere electrodes using an in situ catalytic graphitization-activation strategy. The optimized graphitized and activated potato starch-derived carbon microsphere (GASC10) electrode showed high specific capacitance (198 F/g), excellent rate performance, and long-term stability. It also exhibited a high energy density of 14.67 Wh/kg and a power density of 4142.80 W/kg, demonstrating the importance of controlling graphitization and pore structure for enhanced energy storage. Except pyrolysis, hydrothermal carbonization is widely favored for its mild reaction conditions, controllable particle size distribution, and adaptability to various biomass precursors. Guo et al. [69] introduces an eco-friendly method to synthesize Fe_3_C/Fe/graphitic carbon composites using hydrothermal carbonization and potassium ferrate treatment at 800 °C. The Fe_3_C/Fe-graphitic carbon (FC-1-8) achieved a pseudocapacitance of 428.0 F/g at 1 A/g. After removing Fe_3_C/Fe, the graphitic carbon (FC-1-8-HCl) showed a large surface area (2813.6 m^2^/g) and a high capacitance of 243.3 F/g at 1 A/g, outperforming amorphous carbon electrodes.

In supercapacitor applications, the hierarchical porosity and spherical morphology of 0D biomass-based carbon materials play a crucial role in enhancing electrochemical performance. The spherical shape reduces ion diffusion distance, enabling fast charge–discharge cycles, while the micro/mesopores improve the material’s specific capacitance and cycling stability by facilitating efficient ion transport [64]. Additionally, the low-cost, scalable production processes for biomass-derived carbon nanospheres, such as hydrothermal treatment and spray drying, make these materials highly suitable for large-scale industrial applications.

### 4.2. One-Dimensional Biomass-Derived Carbon Materials

**Figure 4:**
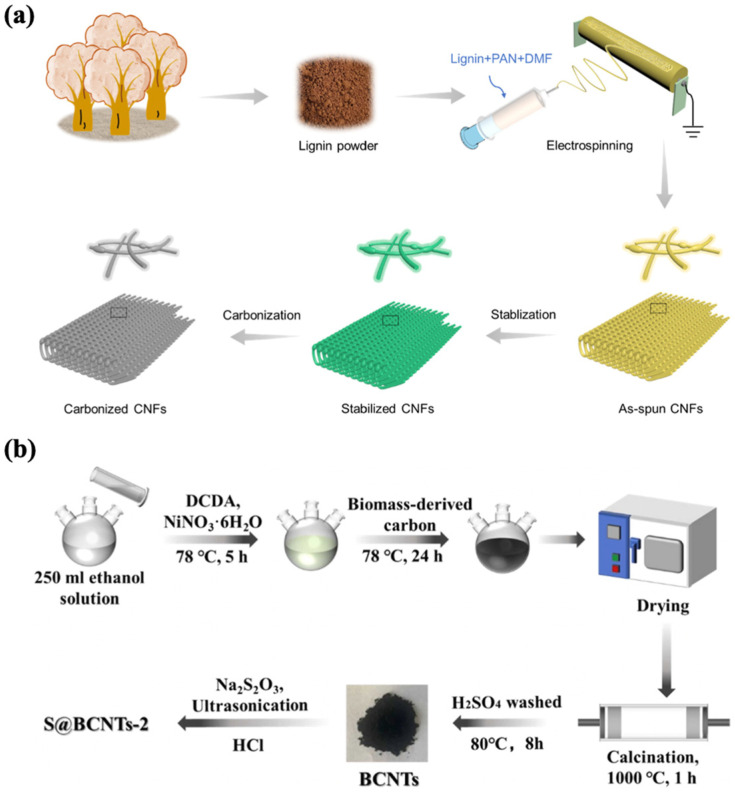
(**a**) The synthesis process of lignin-based carbon nanofibers [86]. (**b**) Schematic illustration of the synthetic processes of bamboo-derived bamboo-like carbon nanotubes (BCNTs) and S@BCNTs-2 [73].

One-dimensional materials are structures with a nanoscale diameter but an extended length, giving them a high aspect ratio. These materials, such as carbon fibers and carbon nanotubes (CNTs), provide continuous pathways for efficient electron transport and rapid ion diffusion along their elongated structures. In the context of biomass-derived carbon materials, 1D structures are often synthesized through methods like electrospinning, which allows for the formation of nanofibers from biomass precursors, followed by carbonization. The linear geometry of 1D materials not only improves electrical conductivity but also enhances mechanical flexibility and structural stability, making them suitable for high-power and durable supercapacitor applications. One-dimensional biomass-derived carbon materials have garnered significant attention for their unique structural and electrochemical properties, making them highly promising for supercapacitor electrode applications (Figure 4). These materials are primarily derived from natural biomass sources rich in fibrous structures, such as wood, lignin, cellulose, bacterial cellulose, and algae. The inherent 1D morphology of these materials provides excellent mechanical stability, high flexibility, and superior electrical conductivity, all of which are crucial for enhancing supercapacitor performance [36].

Carbon nanofibers (CNFs) are fibrous carbon materials with diameters ranging from 10–500 nm and lengths of between 0.5 and 100 μm. Their one-dimensional, interconnected fiber network enables efficient electron transport and rapid ion diffusion, which significantly improves the charge/discharge rate and electrochemical stability in supercapacitors. For example, lignin-based CNFs have been synthesized through an electrospinning process combined with carbonization, achieving highly porous structures with a high specific surface area of up to 837 m^2^/g [74]. The electrospinning technique allows precise control over the pore structure and morphology, which enhances the surface area available for charge storage. Additionally, self-supported ultra-microporous CNFs fabricated from lignin through electrospinning and stabilization processes exhibit abundant micropores that shorten ion transport pathways, further improving the rate capability of the material [86]. Moreover, biomass-derived CNFs can be doped with heteroatoms such as nitrogen (N), boron (B), and sulfur (S), which further enhance their electrochemical properties. For instance, nitrogen-doped carbon nanofibers derived from chitin exhibit a cross-linked fiber structure with hierarchical porosity, providing fast ion transport and high specific capacitance [75]. Similarly, B, N, and F tri-doped lignin-based CNFs synthesized through electrospinning and pyrolysis processes deliver excellent rate performance and energy density due to their improved wettability and surface reactivity [87].

Biomass-derived CNTs have emerged as promising materials for supercapacitor applications due to their unique structural and electrochemical properties. Liu et al. synthesized bamboo-derived bamboo-like carbon nanotubes (BCNTs) with diameters ranging from 50 nm to 300 nm, featuring structural intervals that facilitate ion and electron transport, which are critical for enhancing charge storage and electrochemical performance in supercapacitors [73]. Nitrogen-doped CNTs derived from the biomass of Typha orientalis were fabricated through carbonization and molten salt methods, achieving a diameter of approximately 150 nm, a specific surface area of 281.3 m^2^/g, and a total pore volume of 0.22 cm^3^/g. These features significantly improve ion storage capacity and charge transfer, making them suitable for energy storage devices [88].

Advantages of 1D materials in supercapacitors include excellent mechanical stability, high flexibility, and efficient ion and electron transport. However, their fabrication can be complex and costly, and achieving large-scale production with consistent quality remains a challenge.

### 4.3. Two-Dimensional Biomass-Based Carbon

**Figure 5:**
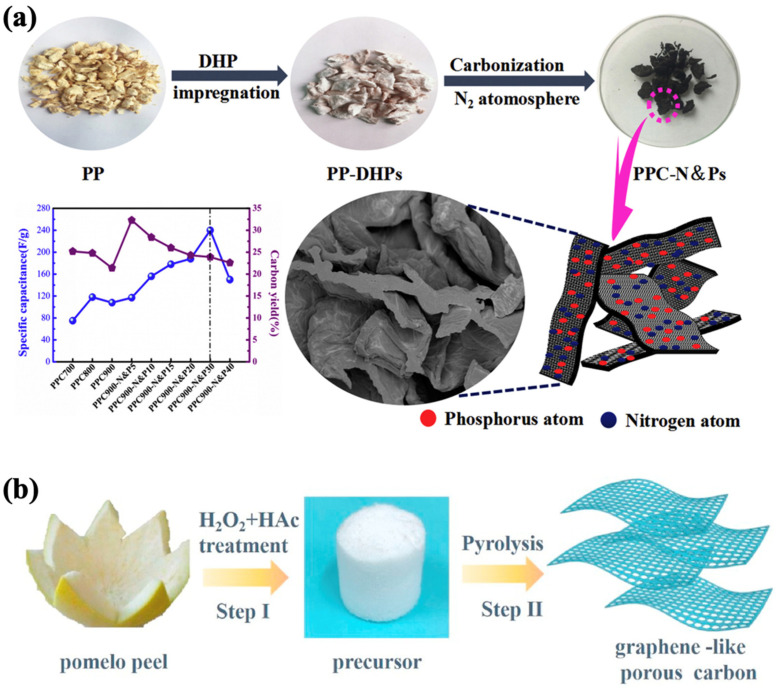
(**a**) Schematic illustration for the synthesis process of porous activated carbon microsheets dual-doped with nitrogen and phosphorus elements (PPC-N and Ps) and its specific capacitance [77]. (**b**) Synthetic route of graphene-like porous carbon nanosheets [93].

Two-dimensional materials are characterized by their sheet-like morphology with nanoscale thickness and extended lateral dimensions. These materials, such as graphene and graphene-like nanosheets derived from biomass, offer large surface areas for ion adsorption and active edges for pseudocapacitive reactions [89]. Biomass-derived 2D materials are often prepared through direct pyrolysis of layered biomass precursors or via template-assisted methods to achieve thin, planar structures. The thinness of 2D materials reduces ion diffusion resistance and improves charge storage capabilities, making them ideal for applications requiring high energy density and fast charge–discharge rates. Biomass-derived 2D carbon materials, characterized by their layered porous structures, heteroatom doping, and defect-rich surfaces, have emerged as promising candidates for high-performance supercapacitors [90]. These materials offer several advantages, including high specific surface area, enhanced conductivity, abundant active sites, and adjustable porosity, which collectively contribute to their excellent electrochemical properties [36]. Unlike traditional graphene, which suffers from high production costs and self-aggregation, 2D carbon materials derived from renewable biomass present a sustainable and cost-effective alternative [91]. The synthesis of biomass-derived 2D carbon materials typically involves environmentally friendly methods such as hydrothermal treatment, freeze-drying, and chemical activation. For instance, pomelo peel has been utilized to prepare nitrogen and phosphorus-doped 2D carbon sheets via a carbonization process, yielding materials with a uniform sheet thickness of 0.3 μm and a porous structure [77] (Figure 5a). These features not only enhance the electrolyte contact area but also facilitate rapid ion transport, leading to improved rate performance and cycling stability in supercapacitors. Similarly, pine nut shells activated with KOH and melamine have been used to fabricate 2D carbon sheets with optimized pore size distribution, contributing to superior ion storage and charge transfer capabilities [92].

In addition, graphene-like 2D nanosheets derived from biomass have demonstrated exceptional potential in supercapacitor applications. For example, graphene-like porous carbon nanosheets prepared from pomelo peels via hydrothermal treatment with acetic acid and hydrogen peroxide exhibit tunable thickness and porosity (Figure 5b) [93]. The incorporation of nitrogen dopants and defects further enhances their specific capacitance by increasing the density of active sites and improving conductivity. Another noteworthy approach involves the direct exfoliation of natural biomass, such as wheat straw, to synthesize layered graphene-like structures [78]. Using hydrothermal and high-temperature graphitization processes, researchers have successfully fabricated carbon sheets with high graphitization degrees, uniform mesoporous structures, and excellent mechanical stability. The hierarchical porosity and layered structure of these materials provide multiple benefits for supercapacitor applications. The thin, layered configuration shortens ion diffusion pathways, enabling faster charge/discharge cycles, while the open edges and interlayer spacing expose more active sites for electrolyte interaction. For example, chitosan-derived carbon nanosheets prepared through freeze-drying and carbonization achieve a specific surface area of over 1000 m^2^/g, with interconnected mesopores that enhance ion transport and storage. These properties significantly improve the energy and power density of supercapacitors [94]. In addition, Wang et al. demonstrates the use of mononuclear anthraquinone derivatives and porous lignin-based graphene oxide (PLGO) in self-assembled colloidal gel electrodes, which are printed as flexible micro-supercapacitors (FMSCs). The FMSCs, with high capacitance (484.8 F/g) and excellent cycle stability (>10,000 cycles), offer a high areal capacitance (43.6 mF/cm^2^) and impressive energy/power densities [95].

While 2D biomass-derived carbon materials exhibit excellent performance in supercapacitors due to their large surface area and efficient ion transport properties, the complex and costly preparation processes can hinder their widespread application. To fully capitalize on the advantages of 2D carbon materials, future advancements should focus on developing more cost-effective and scalable synthesis methods, making them more accessible for large-scale energy storage applications. Additionally, multi-step preparation processes can influence the dimensions and purity of the resulting carbon materials. Each processing step, such as chemical activation or thermal treatment, can alter the structural integrity and introduce impurities, potentially affecting the performance of the final product. Therefore, optimizing these processes is crucial to maintain the desired two-dimensional structure and minimize impurity incorporation.

### 4.4. Three-Dimensional Biomass-Based Carbon

Three-dimensional materials possess interconnected porous networks that provide hierarchical porosity and continuous pathways for ion and electron transport. Examples include carbon foams, aerogels, and 3D porous carbons derived from biomass. These materials are typically synthesized through template-assisted methods, self-assembly processes, or chemical activation techniques that enhance pore formation and surface area. The hierarchical structure of 3D materials allows for the simultaneous optimization of ion accessibility, charge storage, and mechanical stability, enabling their use in high-performance supercapacitors with improved energy and power density. Biomass-derived 3D carbon materials have gained significant attention in supercapacitor applications due to their unique hierarchical porous structures, high specific surface area, and excellent electrical conductivity. These materials provide well-interconnected pores that serve as continuous ion transport channels, enabling faster ion diffusion and charge transfer, thereby improving the overall electrochemical performance of supercapacitors.

**Figure 6:**
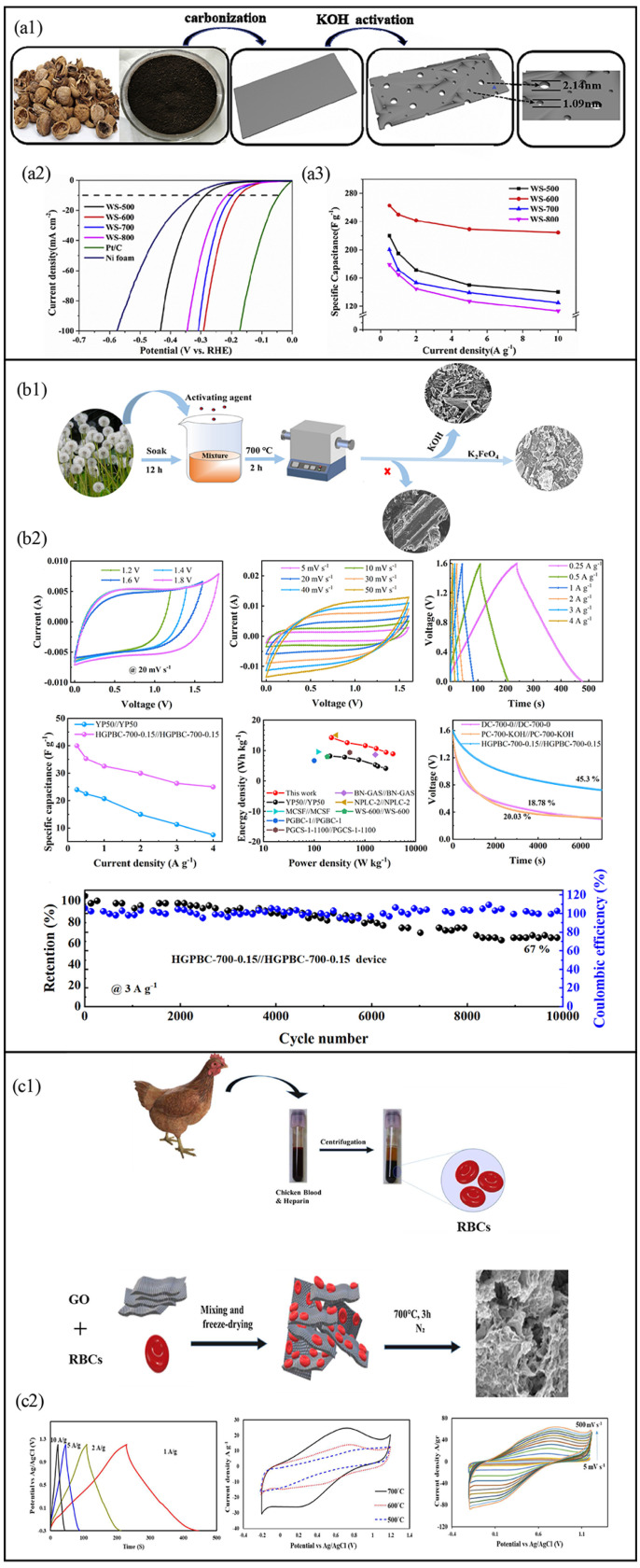
(**a1**) Synthetic method of walnut shell-derived hierarchical porous carbon; (**a2**) LSV curves and (**a3**) specific capacitance of different precipitants synthetic of walnut shell. (WS)-X samples [96]. (**b1**) Illustration of the preparation procedure of carbon materials and (**b2**) electrochemical performances of the high graphitic porous biomass carbon (HGPBC)-700-0.15//HGPBC-700-0.15 supercapacitor device in 0.5 M Li_2_SO_4_ electrolyte [97]. (**c1**) The steps of the preparation of graphene oxide–red blood cells (GO-RBCs) composite and (**c2**) electrochemical performance characterization of GO-RBCs-F-P [100].

For example, Fu et al. synthesized 3D hierarchical porous carbon from walnut shells using KOH activation, achieving a specific surface area of 1037.31 m^2^/g and a micropore volume of 0.51 cm^3^/g (Figure 6a) [96]. This structure reduced ion transport resistance and enhanced charge storage, making the material suitable for energy storage applications. Similarly, Tan et al. prepared highly graphitic porous biomass carbon (HGPBC) from dandelion through a one-step activation–carbonization process using K_2_FeO_4_ (Figure 6b). The resulting flake-like 3D structures facilitated ion transport and electron transfer, leading to improved supercapacitor performance [97]. The poly methyl methacrylate (PMMA) templated NiCo_2_O_4_ material showed a 50-fold increase in surface area compared to the blank, with a well-developed mesoporous structure. This enhancement led to a significant improvement in electrochemical performance, with the specific capacitance reaching approximately 160 F/g. The increased surface area and mesoporosity were key factors in boosting the material’s performance, making it highly suitable for supercapacitor applications [98]. In addition, nitrogen doping in carbon materials significantly enhances their electrochemical performance in supercapacitor applications. For instance, honeycomb nitrogen-doped porous carbon (NPC) synthesized using glucose and trans-1,2-cyclohexane diamine tetraacetic acid as carbon and nitrogen sources, respectively, demonstrated a high specific capacitance of 423 F/g at 0.5 A/g. Additionally, the symmetric supercapacitor based on this NPC exhibited a high energy density of 58.75 Wh/kg and a power density of 250 W/kg, indicating the effectiveness of nitrogen doping in enhancing supercapacitor electrode materials [82]. Minakshi et al. studied eggshell-derived carbon materials and found that their 3D structure significantly enhances ion diffusion while increasing the surface area for charge storage. This improvement leads to higher energy and power densities. The interconnected pore network also contributes to better conductivity and long-term stability, resulting in impressive capacitance retention over 1000 cycles [99].

Another innovative approach involved using chicken red blood cells to construct graphene-like macroporous hybrids (GO-RBCs-F-P) (Figure 6c). These hybrids exhibited a continuous carbon network with interconnected macro-and mesopores (7–22 nm) that promoted rapid electron and ion transport [100]. Similarly, sponge-shaped carbon derived from chicken foot-based collagen demonstrated an efficient 3D porous structure that enhanced electrolyte accessibility and reaction kinetics. For instance, 3D carbon frameworks constructed by interconnected nanocages exhibit high specific surface areas and hierarchical porosity, leading to ultrafast charge/discharge rates and high energy-power densities in supercapacitor applications [101]. Yang et al. demonstrates that 3D porous cellulose sponge carbon (CSC) is an effective anode material for microbial fuel cells (MFCs). The carbonization temperature greatly impacts the material’s properties, with higher temperatures result in lower charge transfer resistance and higher power density. The CSC-700 anode exhibited the highest power density (3.62 ± 0.11 W/m^2^) among lignocellulose-based anodes, while the CSC-1100 anode achieved the highest biomass and power density (4.1 ± 0.1 W/m^2^), showing the promising potential of 3D porous carbon in sustainable energy devices [102].

Aerogels, as a subset of 3D carbon materials, have also shown great promise. Xia et al. fabricated flexible electrodes with outstanding mechanical properties using wood-derived carbon fiber-reinforced cellulose nanofiber/multi-walled carbon nanotube hybrid aerogels [103]. These aerogels provided a robust 3D nanostructure with mesopores, allowing high ion accessibility while maintaining excellent electrical conductivity. In addition, 3D-printed carbon aerogel microlattices have been designed with tunable thicknesses to achieve high areal capacitance. The programmability of these 3D structures allows for the optimization of electrochemical performance, demonstrating the versatility and effectiveness of 3D carbon materials in enhancing supercapacitor electrodes [104].

In summary, biomass-derived 3D carbon materials offer unparalleled advantages for supercapacitor applications, including enhanced ion transport, high specific surface area, and hierarchical porous structures. By optimizing synthesis methods such as KOH activation, salt templating, and doping strategies, these materials achieve superior electrochemical performance, demonstrating their potential as next-generation energy storage solutions.

## 5. Conclusions and Future Work

Biomass-derived carbon materials have demonstrated significant potential for advancing supercapacitor technology due to their renewable nature, tunable properties, and dimensional versatility. This review has highlighted the unique advantages of different carbon material dimensions—0D carbon spheres, 1D fibers and nanotubes, 2D nanosheets, and 3D porous networks—in enhancing charge storage, ion diffusion, and electron transport. Preparation methods such as pyrolysis, gasification, HTC, chemical activation, and physical activation have been instrumental in tailoring these materials to meet the specific demands of supercapacitor applications. By leveraging their hierarchical porosity, high specific surface areas, and excellent conductivity, biomass-based carbon materials offer sustainable and high-performance solutions for next-generation energy storage systems.

Despite significant progress in the application of carbon-based materials in supercapacitors, there remain areas that require improvement and enhancement. Future work can focus on the following aspects. First, there is a need to develop scalable and cost-effective synthesis methods that ensure the consistent quality and performance of carbon materials. Second, understanding the relationship between biomass precursor composition, dimensional structure, and electrochemical properties is critical for optimizing material design. Furthermore, integrating heteroatom doping strategies with advanced structural designs could further enhance ion transport and charge storage capabilities. Additionally, combining biomass-derived carbon materials with other functional materials, such as conductive polymers or metal oxides, may unlock synergistic effects for higher energy density and improved cycling stability. Lastly, addressing the environmental impact of production processes and ensuring sustainable development will be vital for the large-scale adoption of biomass-derived carbon materials in energy storage technologies. Furthermore, building on recent advances, there should be a focus on integrating supercapacitors with green energy devices and developing flexible supercapacitor designs for diverse applications, which will play a critical role in expanding the scope of supercapacitor technology [105]. These advancements will contribute to the realization of efficient, low-cost, and environmentally friendly supercapacitor systems.

## Figures and Tables

**Table 1:** Comparison of biochar production methods.

	Pyrolysis	Gasification	Hydrothermal Carbonization
Feedstocks	Dry biomass (e.g., sawdust, rice husks)	High-ash biomass (e.g., sludge, digestates)	Wet biomass (e.g., food waste, yard waste)
Temperature	300–800 °C	>700 °C	180–250 °C
Atmosphere	Oxygen-limited	Gasifying agents (air, CO_2_, steam)	Subcritical water
Pressure	Ambient	Varies (atmospheric to high)	2–10 MPa
Yield	Moderate	Low	High
Porosity	Moderate, improves with temperature	High microporous/mesoporous structure	Low, requires further activation
Carbon Content	High, increases with temperature	High aromatic content	Moderate
Functional Groups	Moderate	Fewer functional groups	Rich oxygenated functional groups
Graphitization degree	High	Moderate	Low

**Table 2:** Electrochemical properties of 0D to 3D biomass-derived carbon materials.

Carbon Structure and Name		Preparation Method	Specific Surface Area (m^2^/g)	Activation Type	Capacitance Retention (%)	Cycling Stability	Ref
0D	Corncob lignin (HSC-1000)	Spray-drying	1261.7	NaOH	64.2	5000	[65]
	Potato starch-based carbon microspheres (GASC10)	Pyrolysis	2369	KOH	83.3	1000	[66]
	Rape pollen hierarchically porous carbon (RPHPC-750)	Pyrolysis	748.6	ZnCl_2_+FeCl_3_	94.5	20,000	[67]
	Ganoderma lucidum spores (Spore-800-NH_3_)	Pyrolysis	408.45	N-doped	93.3	1000	[68]
	Glucose-derived carbon (FC-1-8-HCl)	Hydrothermal	2813.6	K_2_FeO_4_	51.7	10,000	[69]
1D	Graphitic carbon microtubes (PGCMT)	Pyrolysis+graphitization	1066.56	K_4_Fe(CN)_6_	61.8	5000	[70]
	Cellulose tubes	Hydrothermal	808.25	KOH	/	>300	[71]
	Iron complex nanoparticles encapsulated into biomass-derived N, P-codoped carbon nanotubes (FeX@NPCNTs)	Hydrothermal	566.07	N+P-doped and K_4_Fe(CN)_6_	more than 99	50,000	[72]
	Bamboo-structure carbon nanotubes (S@BCNT-2)	Pyrolysis	437.41	N doped+ Na_2_S_2_O_3_	Close to 100	>500	[73]
	Biomass-based carbon fibers (CFs-5)	Pyrolysis	837.40	H_3_PO_4_	77.3	/	[74]
	N self-doped carbon nanofibers (CChCNs)	Pyrolysis	377.31	/	74.5	500	[75]
2D	Loofah sponge (PCNs)	Pyrolysis	18.7	/	>80	1200	[76]
	Pomelo peel -derived carbon microsheets (PPC900-N and P30)	Pyrolysis	807.7	N+P doped	Approximately 100	10,000	[77]
	Wheat straw graphene	Hydrothermal	35.5	KOH	92.4	300	[78]
	Peanut shell activated carbon	/	525.68	KOH	98	100	[79]
3D	Glucose hierarchically porous carbon materials (HPC)	Pyrolysis	1806	Zn(NO_3_)_2_	97.5	5000	[80]
	Glucose derived (CNS/CF_0.15_)	Pyrolysis	1068	K_3_ [Fe(C_2_O_4)3_]·H_2_O	96.8	10,000	[81]
	Honeycomb nitrogen-doped porous carbon (NPC)	Hydrothermal	1946.8	KOH	92.5	5000	[82]
